# Intravenous immunoglobulin treatment of the post-polio syndrome: sustained effects on quality of life variables and cytokine expression after one year follow up

**DOI:** 10.1186/1742-2094-9-167

**Published:** 2012-07-09

**Authors:** Henrik Gonzalez, Mohsen Khademi, Kristian Borg, Tomas Olsson

**Affiliations:** 1 Division of Rehabilitation Medicine, Department of Clinical Sciences, Karolinska Institutet, Danderyd Hospital, blg 39, fl 3, S-192 88, Stockholm, Sweden; 2 Neuroimmunology Unit, Department of Clinical Neuroscience, Karolinska Institutet, Stockholm, Sweden

**Keywords:** Post-polio syndrome (PPS), Intravenous immunoglobulin (IVIG), Cerebrospinal fluid (CSF), Inflammatory cytokines

## Abstract

**Background:**

Expression of inflammatory cytokines in cerebrospinal fluid (CSF) has led to the hypothesis of intrathecal chronic inflammation to explain the denervation observed in post-polio syndrome (PPS). It has been shown that therapy with intravenous immunoglobulin (IVIG) improves physical performance and dampens down the inflammatory process at 6 months in PPS patients. We here examined the effects of IVIG on cytokine expression and clinical outcome one year after IVIG treatment.

**Methods:**

From a previous study with 135 PPS patients included, 41 patients were further evaluated before un-blinding for one year (21 placebo and 20 treated with IVIG, Xepol® 50 mg/ml), and were assessed for clinical variables by performing the Short Form-36 survey (SF-36) questionnaire assessment, the 6 minute walk distance test (6MWT) and registering pain level by Visual Analogue Scale (VAS) after IVIG treatment. A separate cohort of 37 PPS patients went through lumbar puncture (LP) at baseline and 20 patients, treated with IVIG, repeated the LP one year later. Thirty patients affected with other neurological diseases (OND) were used as control group. Inflammatory cytokines TNF, TGFβ, IFNγ, IL-23, IL-13 and IL-10 were measured in blood cells and CSF cells with RT-PCR.

**Results:**

Scores of the physical components of SF-36 were significantly higher at the one year follow up time-point in the IVIG-treated patients when compared to baseline as well as to the control subjects. Pain VAS score and 6MWT improved significantly in the IVIG-treated patients when compared with baseline Relative expression of TNF and IFN-γ in both PBMCs and CSF from PPS patients were increased compared to OND subjects at baseline (p < 0.05). One year after IVIG-treatment a decreased expression of IFN-γ and IL23 was found in CSF of PPS patients, while anti-inflammatory IL-13 was increased (p < 0.05).

**Conclusions:**

IVIG has effects on relevant QoL variables and inflammatory cytokines up to one year in patients with PPS. This gives a basis for scheduling IVIG in upcoming trials with this therapy.

## Background

The post-polio syndrome (PPS) may appear several decades after an acute poliomyelitis. The late symptoms characterizing PPS are increased or new muscle weakness, fatigue and pain leading to gait disturbances, breathing difficulties as well as swallowing problems [1-3]. It is estimated that 15-85% of poliomyelitis survivors develop PPS (depending on criteria and population) [1]. As the number of survivors is large, approximately 20,000,000 people world-wide, PPS is one of most common motor neuron diseases [3]. Current diagnosis is based on thorough clinical examinations in order to eliminate other possible diagnosis. Once a diagnosis has been made, treatment options can be considered, which so far mainly has been various forms of rehabilitation and physiotherapy.

In PPS, there is an ongoing denervation which may explain the increased weakness in muscles earlier affected by poliomyelitis [4,5]. The denervation is compensated by collateral sprouting leading to an increase of the area of the motor units. However, reinnervation cannot sufficiently compensate for denervation leading to whole or partial loss of motor units followed by a decrease of muscle strength [4]. It is debated whether this deterioration is caused by further loss of motor neurons due to normal aging, genetics, deleterious over-use of remaining motor neurons, and/or an active disease process, perhaps involving chronic intrathecal inflammatory damage [6-10]. These observations are supported by our and others’ findings of and increased expression of pro-inflammatory cytokines particularly in the intrathecal compartment [11-15]. The driving force of this inflammation has remained unclear. However, if the deterioration is indeed driven by inflammation, it should be accessible for therapy.

In a series of studies we have explored if intravenous immunoglobulin (IVIG) treatment can affect the clinical condition and signs of inflammation. This treatment modality was chosen because of the pattern of the inflammatory process and since it has been reported to be beneficial in other neuro-inflammatory conditions with a good safety profile. A dampening down of the pro-inflammatory cytokines was found when IVIG was administered [13] and assessment of clinical variables in this cohort showed beneficial effects [16]. We then performed a double blind placebo controlled study with clinical endpoints evaluated 6 months after treatment. The study revealed significant effects on muscular strength, physical activity, and quality of life (QoL) regarding vitality and pain at six months after treatment [17]. A smaller placebo controlled study and a recently performed open study have documented significant effects on pain [14,18]. However, the analysis of cytokine expression profile has been limited to few patients and restricted sets of cytokines, limiting the options of any correlations to clinical disease variables.

Widespread recommendation for IVIG use in PPS would require replication in independent studies, which would need to consider dosing intervals. While most chronic neuro-inflammatory diseases other than PPS require several courses of IVIG each year, at relatively high cost, our clinical impression is that IVIG in PPS cause improvements over longer periods, and that quite infrequent dosing would be necessary. Thus, on the basis that pro-inflammatory cytokines are dampened down 2–3 months [15], and knowing that effects on clinical variables are present 6 months after treatment [17], we now evaluated the IVIG treatment effects on these measures one year after treatment in an extended set of patients. Such data would give a basis for choosing intervals of repeated dosing of the drug.

## Methods

### Study design

A clinical study extension from the previous double blind placebo controlled trial in which 135 PPS patients were evaluated for clinical endpoints 6 months after treatment [17], was performed. A cohort of 41 patients from one of the 4 participating centers in the previous study (the Danderyd Hospital), were recruited and further evaluated, before un-blinding, one year later. They were asked to perform the SF-36 Questionnaire, the 6 minute walk distance test and pain status evaluation according to the Visual analogue scale (VAS). After breaking of the trial code (un-blinding) it turned out that this group consisted of 20 IVIG treated patients (mean age 61.7 years, range 52–75, 70% females) and 21 from the placebo group (mean age 61.9 years. range 46–75. 57.1% females). Lumbar puncture (LP) was not included in the protocol of the original study. Thus, this study was complemented with a cytokine expression analysis including 37 patients from the placebo-treated group of the previous trial [17], 17 of them dropped out and 20 completed the study one year later. Thirty patients affected with other neurological diseases (OND) (mean age 41.4 years, range 24–61, 70% females) going through neurologic diagnostic work up were used as control group. The diagnosis of the OND group were as follow: Ependymoma (n = 1), Dissociative syndrome (n = 3), Cerebral infarction (late effect) (n = 1), Migraine (n = 1), Neurastenia (n = 1), Neuromuscular bladder disturbance (n = 2), Psychosis (n = 5), Sensory symptoms (n = 3), Spinal stenosis (n = 2), Tension headache (n = 3), Vertebral dissection (n = 1), Vertigo (n = 3), Paresthesia (n = 4). Cytokine expression was evaluated through relative quantization of mRNAs by real-time quantitative RT-PCR. Selected cytokines for analysis were: pro-inflammatory, Th1 pathway-related such as tumor necrosis factor (TNF) and interferon-γ (IFN-γ); Th17 pathway-related such as IL-23; and anti-inflammatory, Th2 pathway-related such as IL-10 IL-13, and TGF-β.

**Figure 1:**
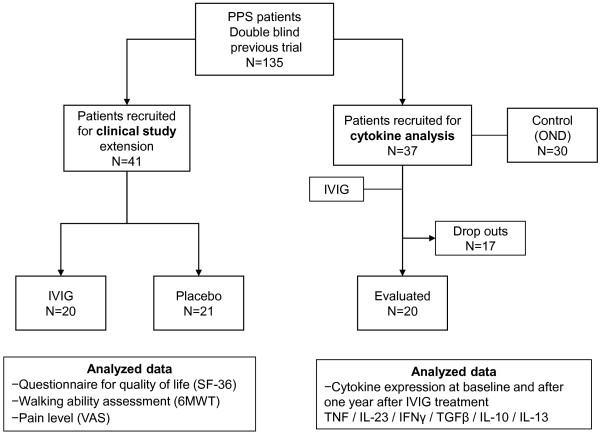
Flow of patients through the study and data available for analysis.

Flow of patients and data available for analysis are shown in Figure 1.

### Study subjects

For the clinical study extension, patients were diagnosed and included according to previously reported criteria [17,19]. Included patients had been randomized to either intravenous infusion of IVIG (Xepol™ human immunoglobulin 50 mg/ml solution for intravenous infusion, batch number IAGD1HDHC1, Instituto Grifols S.A., Barcelona, Spain) or placebo consisting of solution in bottles of identical size and appearance (Instituto Grifols S.A., Barcelona, Spain). A total of 90 g of IVIG was given and repeated 3 months later. The placebo solution contained glucose and water for injection. The dosage levels and infusion periods are described elsewhere [17].

For the cytokine expression study, PPS patients were subjected to venous and lumbar puncture before the administration of a single dose of IVIG (90 g) to perform the analysis of cytokines in peripheral blood mononuclear cells (PBMCs) and the CSF. One year after IVIG infusion, 20 patients out of the 37 recruited volunteered for another blood test and lumbar puncture. Blood and CSF samples of patients with OND (n = 30) were obtained from the in-house biobank at Karolinska University Hospital containing samples collected during routine neurological diagnostic work-up. All enrolments to the study followed the recommendations of the declaration of Helsinki and the study was approved by the Ethics committee of the Karolinska Institutet. Oral and written information was given to the patients and confirmed consent in writing was received before inclusion.

### Short form-36 survey

The Short Form-36 survey (SF-36) is a widely used and extensively validated QoL instrument that contains 36 items that assess patients’ health status and its impact on their lives [20,21]. The SF-36 is composed of eight multi-item scales (Physical Functioning, Role-Physical, Bodily Pain, General Health, Vitality, Social Functioning, Role-Emotional, Mental Health), with scores ranging from 0 to 100. Higher scores indicate higher QoL.

Data from the instrument can also be used to calculate two summary scores, the Physical Component Summary (PCS) and the Mental Component Summary (MCS). These summary scales are computed as standardized scores such that a mean score of 50 corresponds to that of the general US population. For the PCS and MCS scales, a change of 5.0 points was considered a clinically meaningful difference.

The psychometric performance of the SF-36 has been well established across a wide variety of disease states [22-26]. The language versions used in the studies were obtained from the developer and were administered as patient-completed paper-and-pencil instruments with no allowance for proxy completion.

### Walking ability assessment

Walking ability was measured by the 6-minute walk test (6MWT) [27,28]. The subjects were asked to walk a measured distance (25 meters) back and forth at an individually chosen speed for 6 minutes. Assistive devices and walking aids were used if this was the standard situation for the patient and, if so, were registered so the second test could be performed under the same conditions. A longer distance is suggestive of increased walking ability.

### Pain assessment

The Visual Analogue Scale (VAS) [29] was used to evaluate the pain level. It consists of a 100-mm scale where 100 mm stands for the worst imaginable pain and zero stands for no pain.

### Blood and CSF sampling

Paired CSF and blood samples were available. Peripheral blood (PB) was sampled in sodium citrate-containing cell preparation tubes (Vacutainer CPT, Becton Dickinson and Company, USA) and CSF in siliconized glass tubes or polypropylene tubes. CSF samples were immediately centrifuged and the pellet was recovered and stored at −80 °C. PBMCs were separated by density gradient centrifugation. Cells from the interphase were collected and washed twice with Dulbecco´s PBS. The proportion of viable cells was assessed by trypan blue exclusion. Finally, the cells were pelleted, frozen on dry ice and stored at −80 °C until use.

### Relative quantitation of mRNAs by real-time quantitative RT-PCR

Cell pellets were lysed and total RNA was extracted (PicoPure RNA isolation kit, Arcturus Bioscience Mt. View, CA). Samples were incubated with DNAase (Qiagen RNAase free DNAase set, Hilden, Germany) for 15 min. at RT in order to avoid amplification/detection of contaminating genomic DNA. Reverse transcription was performed with 10 μl total RNA (5–10 ng), random hexamer primers (0.1 μg; Gibco BRL) and Superscript Reverse Transcriptase (200 U; Gibco BRL). Real-time PCR was performed using a BioRad iQ5 iCycler Detection System (BioRad Laboratories, Ltd) with a three-step PCR protocol (95 °C for 10 min. followed by 50 cycles of 95 °C for 15 sec., 60 °C for 30 sec. and 72 °C for 30 sec.), and with SYBR green fluorophore. Reactions were performed in a total volume of 15μL including 7.5μL 2x SYBR Green PCR Master Mix (BioRad Laboratories, Ltd), 1.0μL of each primer at 5 μM concentration, 2μL of the previously reverse-transcribed cDNA template and 4.5μL double-destilated milli-Q H_2_O. Primers were designed with the Primer Express Software (Perkin Elmer) in our laboratory. The primer sequences for TNF (Genbank Accession no: NM_000594) are: forward primer, 5´ CCA GGG ACC TCT CTC TAA TCA GC 3´; reverse primer, 5´ CTC AGC TTG AGG GTT TGC TAC A 3´. The primer sequences for IFN-γ (Genbank Accession no: NM_000619) are: forward primer, 5´ GGT TCT CTT GGC TGT TAC TG 3´; reverse primer, 5´ TCT GTC ACT CTC CTC TTT CC 3´. The primer sequences for IL-23 (Genbank Accession no: NM_016584) are: forward primer, 5´ CAA GTG GAA GTG GGC AGA G 3´; reverse primer, 5´ CAG CAA CAG CAG CAT TAC AG 3´. The primer sequences for IL-10 (Genbank Accession no: NM_000572) are: forward primer, 5´ GCT GGA GGA CTT TAA GGG TTA C 3´; reverse primer, 5´ TTG ATG TCT GGG TCT TGG TTC 3´. The primer sequences for IL-13 (Genbank Accession no: NM_002188) are: forward primer, 5´ ATT GCT CTC ACT TGC CTT GG 3´; reverse primer, 5´ CTG GTT CTG GGT GAT GTT G 3´. The primer sequences for TGF-β (Genbank Accession no: NM_003239) are: forward primer, 5´ AAC GAA CTG GCT GTC TGC 3´; reverse primer, 5´ CCT CTG CTC ATT CCG CTT AG 3´. The primer sequences for GAPDH (Genbank Accession no: NM_002046) are: forward primer, 5´AGG GCT GCT TTT AAC TCT GGT AAA3´; reverse primer, 5´CAT ATT GGA ACA TGT AAA CCA TGT AGT TG3´. The primer pairs were tested by temperature gradient (55 °C −65 °C for annealing step) programmed and the PCR products were controlled by an agarose gel to for single band of the expected size of each specific target. Sequencing of the different bands (Cybergene AB, Huddinge, Sweden) confirmed homology with the reported sequences for human VAV1, TNFα and GAPDH respectively. Relative quantitation of mRNA levels was performed using the standard curve method, with amplification of mRNA and GAPDH. The standard curves were created using five serial dilutions (1:10, 1:10^2^, 1:10^3^, 1:10^4^ and 1:10^5^) of cDNA from human blood cells stimulated with lectin (Con A). The samples were run in duplicate with primers against GAPDH and the target mRNA in different wells. Samples without added cDNA served as negative controls. The relative amount of mRNA in each sample was calculated as the ratio between the target mRNA and the corresponding endogenous control GAPDH. The relative expression for each target was then normalized to an arbitrary maximum value of 1.0 represented by the sample with highest expression.

### Statistics

Differences between groups and within groups regarding clinical parameters were analyzed for significance with the Mann–Whitney test and with the non-parametric Wilcoxon´s signed-ranks test (JMP 3.2, SAS Institute Inc., NC), respectively, since data were not supposed to be equally distributed. Differences in relative cytokine mRNA levels for comparing of OND vs PPS were tested for significance with the Mann–Whitney test, and for baseline vs IVIG-follow-up were tested for significance with non-parametric Wilcoxon´s matched-pairs signed-ranks test (JMP 3.2, SAS Institute Inc., NC). Correlations between clinical data and cytokine mRNA levels were analyzed with Spearman´s rank (Sr) test. Analyses were done using the software GraphPad Prism 5.0 (San Diego, CA, USA).

## Results

### Clinical study results

In the 41 patients cohort (20 IVIG-treated and 21 placebo-treated) that was followed in their clinical parameters, the score of the QoL parameter SF-36 Physical Component Summary (PCS) reached statistical significance (p = 0.020) at the one year follow up time-point in favor of the IVIG treated patients and also with respect to the baseline score before treatment (p = 0.021). Similarly, even the sub domain Physical Functioning (PF) was significantly different between the groups (p = 0.028) and with respect to baseline (p = 0.014). These results are summarized in Table 1.

**Table 1:** Clinical parameters analysis (quality of life, QoL) according to the Short Form-36 (SF-36) survey and 6 minute walk test (6MWT), comparing the IVIG-treated and placebo group

	**IVIG(n=20)**			**Placebo(n=21)**			**Between**
**SF-36 score**	**Before**	**1 year**	**p**	**Before**	**1 year**	**p**	**group diff. p**
	**mean (SD)**	**mean (SD)**		**mean (SD**)	**mean (SD)**		
PF	33.2 (17.9)	39.8 (19.2)	0.014	40.7 (22.7)	41.0 (22.0)	0.761	0.028
RP		30.0 (28.8)	42.5 (39.8)	0.240	50.0 (40.3)	48.0 (40.0)	0.757	0.278
BP		50.8 (18.1)	55.5 (21.6)	0.189	55.4 (24.5)	50.7 (27.3)	0.224	0.075
GH		53.1 (22.4)	61.3 (24.2)	0.058	58.1 (22.5)	61.8 (24.2)	0.190	0.290
VT		43.0 (22.6)	49.3 (27.3)	0.267	46.4 (23.8)	47.3 (22.2)	0.948	0.300
SF		66.9 (24.1)	73.8 (26.6)	0.094	70.8 (26.3)	71.4 (23.8)	0.897	0.191
RE		61.7 (43.6)	66.7 (40.5)	0.665	63.5 (43.3)	73.0 (38.9)	0.261	0.736
MH		74.4 (17.9)	76.0 (19.3)	0.652	72.6 (17.2)	72.5 (17.6)	0.881	0.844
PCS		28.9 (6.1)	32.8 (8.2)	0.021	34.0 (10.0)	33.1 (11.3)	0.394	0.020
MCS		48.7 (13.2)	50.0 (12.5)	0.681	47.6 (12.4)	49.3 (11.9)	0.192	0.531
VAS		31(24)	23(20)	0.013	31(22)	29(28)	0.509	0.449
6MWT, m		351 (110)	400 (112)	306 (111)	325 (125)			
	n=19	n=19		n=20	n=20			

SF-36 scores range from 0 to 100. 6MWT are recorded in meters.

Physical Functioning (PF), Role-Physical (RP), Bodily Pain (BP), General Health (GH), Vitality (VT), Social Functioning (SF), Role-Emotional (RE) and Mental Health (MH).

Pain score (VAS) decreased significantly at the one year follow up in the IVIG treated patients (p = 0.013) when compared with baseline. No change in VAS was seen in the placebo group and there was no significant difference between the IVIG and the placebo treated patients at the one year follow up (Table 1).

Walking ability after one year showed a significant improvement in the IVIG treated patients (p < 0.001) but not in the controls. There was no statistical difference after one year follow up between the IVIG and placebo group (Table 1).

### Cytokine analysis results

Of the 37 patients with PPS that were recruited for initial lumbar puncture and subsequent IVIG infusion, mean age was 58.9 years (range 36–76 years), with a 56.8% of females, and 2 out of the 37 patients with oligoclonal bands in CSF. Of the 20 patients that volunteered for the one-year follow up of cytokine analysis, mean age was 62.7 years (range 49–79) with a,75.0% of females. In the 30 subjects of the OND cohort, mean age was 41.4 years (range 24–61 years), with a 70.0% of females, all of them without oligoclonal bands in CSF.

**Figure 2:**
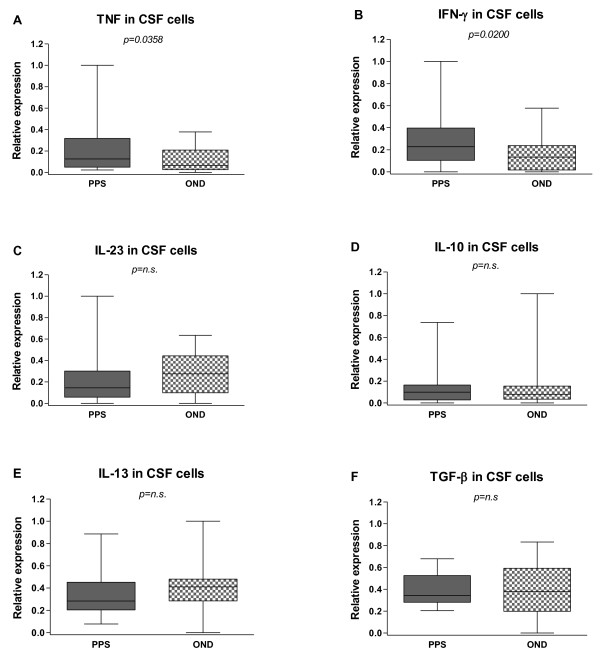
**Baseline levels of inflammatory cytokines in the cerebrospinal fluid (CSF) cells.** Cytokines were measured in patients with Post-polio syndrome (PPS) and with other neurological diseases (OND). The median-based box plots indicate 25th/75th percentiles and whiskers indicate 5th/95th percentiles. Values of statistical significance are indicted (n.s.: non significant).

**Figure 3:**
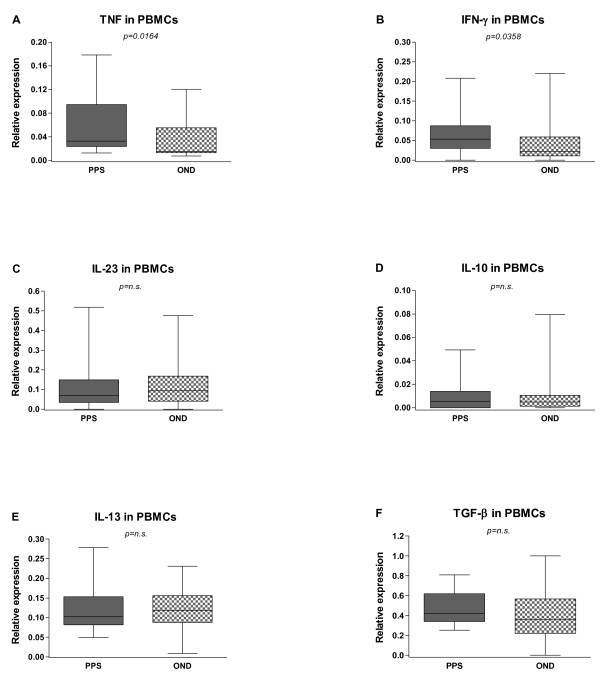
**Baseline levels of inflammatory cytokines in the peripheral blood mononuclear cells (PBMCs).** Cytokines were measured in patients with post-polio syndrome (PPS) and with other neurological diseases (OND). The median-based box plots indicate 25th/75th percentiles and whiskers indicate 5th/95th percentiles. Values of statistical significance are indicted (n.s.: non significant).

**Figure 4:**
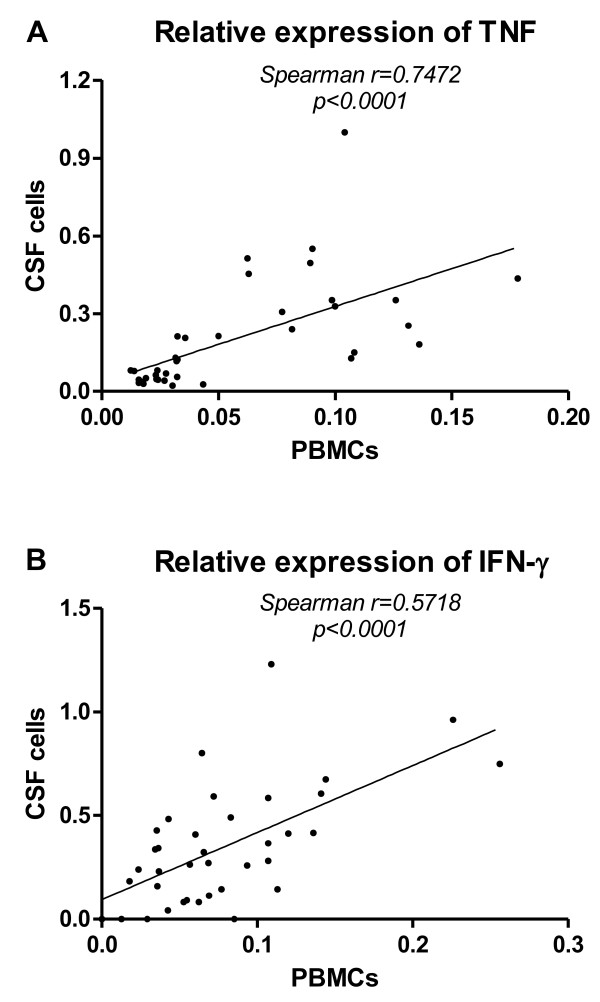
Correlation study of pro-inflammatory cytokines in systemic and intrathecal compartments.

Values of cytokine mRNA expressions in the CSF cells and PBMCs are shown in Table 2. At baseline levels there was a significant increase of mRNA expression for the two pro-inflammatory cytokines TNF and IFN-γ in both CSF (Figure 2A and B) and blood (Figure 3A and B) from PPS treated patients compared to the OND group (TNF: CSF, p = 0.0358; PB p = 0.0164 and IFN- γ : CSF, p = 0.0200; PB p = 0.0358), while none of the other cytokines differed significantly between the groups (Figure 2C–F and Figure 3C–F). For these cytokines the CSF and blood levels correlated strongly (CSF: Sr = 0.7472, p < 0.0001; PB: Sr = 0.5718, p < 0.0001) (Figure 4A and B).

**Table 2:** Levels of cytokine relative expression in the cerebrospinal fluid (CSF) cells and peripheral blood mononuclear cells (PBMCs) in patients with post polio syndrome (PPS) and patients with other neurological disorders (OND) used as control

**Cytokine expression**	**PPS (n=37)**		**OND (n=30)**	
	CSF cells	PBMCs	CSF cells	PBMCs
TNF*	0.204 ±0.0340	0.058 ±0.0071	0.112 ±0.0198	0.034 ±0.0060
IFN-γ*	0.281 ±0.0380	0.063 ±0.0072	0.158 ±0.0292	0.040 ±0.0082
TGF-β	0.391 ±0.0234	0.477 ±0.0275	0.398 ±0.0428	0.403 ±0.0440
IL-10	0.148 ±0.0283	0.009 ±0.0019	0.129 ±0.0367	0.011 ±0.0030
IL-13	0.336 ±0.0307	0.117 ±0.0087	0.426 ±0.0448	0.123 ±0.0092
IL-23	0.238 ±0.0446	0.103 ±0.0177	0.274 ±0.0333	0.125 ±0.0213

Values are expressed as mean ± SEM. * p<0.05 between patient groups and between compartments.

**Figure 5:**
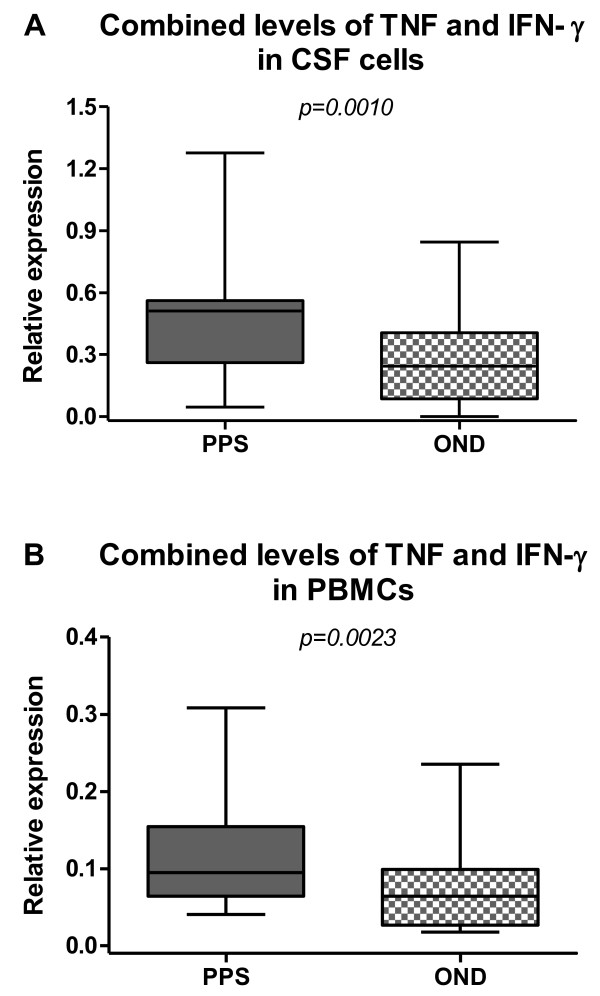
**Combined levels of pro-inflammatory cytokines in the cerebrospinal fluid (CSF) cells and peripheral blood mononuclear cells (PBMCs).** Tumor necrosis factor (TNF) and interferon γ (IFN-γ) were measured in patients with post-polio syndrome (PPS) and with other neurological diseases (OND). The median-based box plots indicate 25th/75th percentiles and whiskers indicate 5th/95th percentiles. Values of statistical significance are indicted (n.s.: non significant).

However, the TNF and IFN- γ levels did not correlate in the same compartment (data not shown). Instead there were many individuals with high TNF but low IFN- γ in the same compartment. This prompted us to add the levels of both cytokines together to visualize a combined form of pro-inflammatory cytokine levels. With this procedure, the discrimination between PPS and OND became more apparent and significant (CSF: p = 0.0010; PB: p = 0.0023) (Figure 5A and B).

**Figure 6:**
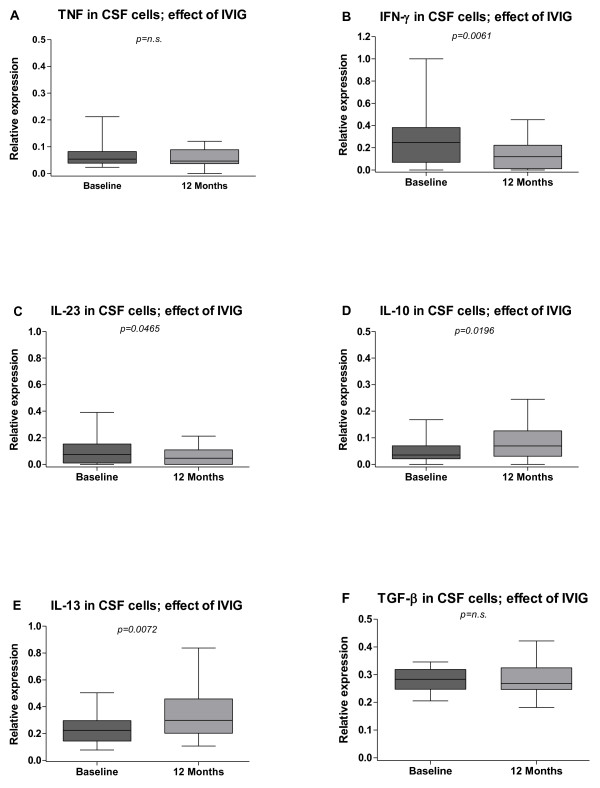
**Effects of intravenous immunoglobulin (IVIG) on levels of inflammatory cytokines in cerebrospinal fluid (CSF) cells.** The median-based box plots indicate 25th/75th percentiles and whiskers indicate 5th/95th percentiles. Values of statistical significance are indicted (n.s.: non significant).

The effect of a single IVIG infusion was a significant decrease of IFN- γ and IL- 23 mRNA expressions in CSF cells (p = 0.0061 and p = 0.0465 respectively) after one-year follow up in patients with PPS (Figure 6B and C). There was a trend towards a sustained decrease for TNF and a significant increase for IL-10 (Figure 6A and D). IL-13 was also significantly increased (p = 0.0072) (Figure 6E). TGF-β did not show any trend after one year follow up (Figure 6F).

**Figure 7:**
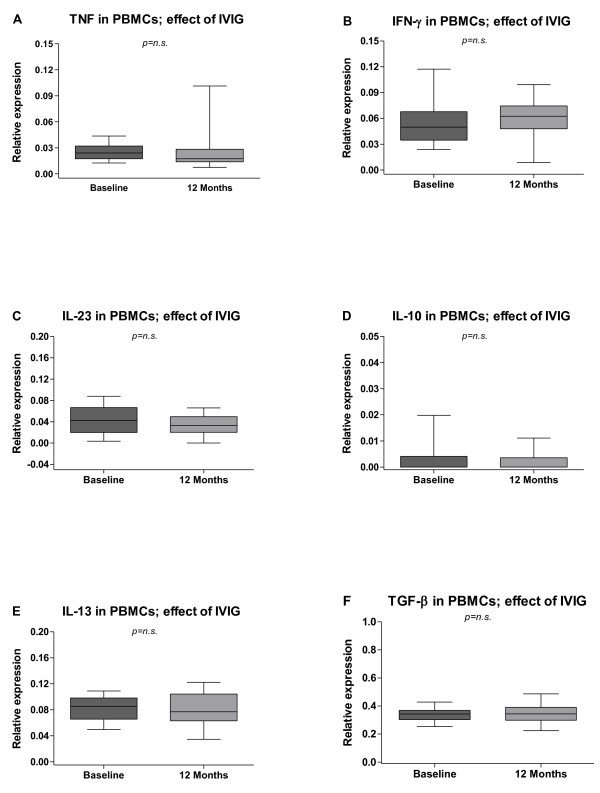
**Effects of intravenous immunoglobulin (IVIG) on levels of inflammatory cytokines in peripheral blood mononuclear cells (PBMCs).** The median-based box plots indicate 25th/75th percentiles and whiskers indicate 5th/95th percentiles. Values of statistical significance are indicted (n.s.: non significant).

In peripheral blood no significant differences in mRNA expressions of cytokines were detected in the follow-up results (Figure 7A–F).

## Discussion

PPS is a relatively slow but a consistently progressive condition [1]. Due to these disease characteristics it is difficult to detect changes in the condition following an intervention in the short-term perspective. Our clinical impression is that following an IVIG treatment the condition has a peak improvement somewhere between 6 months and one year after a single infusion. The results presented here confirm the clinical impression.

Although we did not have the opportunity to follow all the patients in the previous larger study, we had a chance to commit an evaluation of a subgroup of the larger group. As a consequence, there are several limitations of this study. First, the clinical study was performed in a sub-group analysis from one center of the initial trial and so it was not intentionally powered for the analysis presented here. Second, the clinical and cytokine parameters were not evaluated in the same cohort of patients since lumbar puncture was not a part of the protocol of the original study. Thus, the results do not allow for a direct correlation between clinical improvement and in the changes in the CSF cytokine profile within the same cohorts. Nevertheless, since the PPS population was homogeneous because it came from the same previous clinical trial and the objective was to observe IVIG effects after one year follow-up, the results can still be validly discussed.

The clinical parameters at the one-year follow up time-point showed that the physical component of SF-36 was significantly increased in favor of the IVIG treated group. Interestingly, the physical component score (PCS), which was one of the primary variables in the Randomized Controlled Trial, was significantly higher in the IVIG treated group at the one year follow up [17]. Moreover, the vitality sub domain of SF-36, on the contrary to the finding at 6 month [17], did not significantly change between groups after one year follow up. However, pain assessment and walking ability parameters positively evolved from before to after treatment in the IVIG group.

We assume that infusion of IVIG causes a significant increase in QoL for parameters related to physical function. In our opinion, the decreased and surprisingly long lasting effect of a single dose of IVIG has an immediate effect on vitality and pain. One may speculate that the delayed effect on physical parameters could be the result of an enhanced accommodation to routine activity level as well as training and exercise. While in the previous trial [17] the effect on pain could not be excluded as the cause of the positive outcome on motor function, in the present study no significant effect on pain was found in this long term interval. In a recently published study a favorable and statistically significant effect on pain was found in PPS patients 6 months after IVIG treatment [18]. Thus, there is a reason to believe that pain reduction may play a role in the improvement of quality of life for physical function. Further investigation to shed light on these questions is deserved.

To assess the role of inflammation in the same clinical context, in this study we analyzed the cytokine expression profile and immunological variables in blood and CSF in a cohort of PPS patients, one year after IVIG treatment. Consistent with our previous findings [13], but in this report with a new technique and with a larger number of cytokines, we confirm that PPS patients have an active neuro-inflammatory process with increase of Th1 cytokines both in the CSF and to a smaller extent in the PB. Levels of protective cytokines such as IL-10 and IL-13 were similar to those of control patients (OND) before treatment. It is of importance to mention that to exploring the normal subjects with lumbar puncture is not an easy task. For this reason, we have used a non-inflammatory OND cohort as an alternative to healthy subjects against neuroinflammatory conditions such as PPS in this study. The OND cohort used for comparison of baseline cytokine expression to PPS was rather heterogeneous, though care was taken to include persons with non-inflammatory neurological disease. In addition, this type of control subjects have in several previous publications from our group displayed a low expression of cytokines in blood and CSF compared to well accepted inflammatory diseases like multiple sclerosis [30,31]. After treatment with IVIG the Th1 cytokine levels were significantly decreased while the levels of IL-10 and IL-13 were significantly increased. Levels of IL-23 were low in CSF at baseline, and decreased even more one year after a single dose of IVIG. It has been shown that IL-23 participates in the pathogenesis of autoimmune inflammation by promoting the development of a pathogenic CD4^+^ T-cell population that produces IL-6, IL-17 and tumor-necrosis factor (TNF) [32]. As this cytokine promotes auto-inflammatory disease, one year lasting IVIG-induced suppression of IL-23 and IFN-γ levels is of special interest not only for PPS but also for other neurological diseases. However, the fact that changes induced by IVIG occur in the PBMCs as well as the CSF cells may reflect some type of systemic process that cannot be ruled out. CSF is more difficult to obtain and, thus, this finding also suggest that cytokine analysis in PB may be valid for studies of systematic inflammation and potentially for selecting PPS patients for IVIG treatment.

In a recent study Melin et al. (K Borg 2012 personal communication) were able to show an increase of enzymes involved in the prostaglandin pathway in blood vessels of muscle of PPS patients. This may be a background for pain in PPS and may indicate the presence of a systemic process. Further studies are needed to elucidate the role of prostaglandin and the possible effect of IVIG.

## Conclusions

We suggest, on the basis of the data from the present study, that infusion of IVIG causes beneficial changes in the CSF cytokine profiles in PPS patients and that this action is paralleled by a significant increase in QoL for parameters related to physical function that persist even one year after treatment. Our observations of long term sustained effects of IVIG on clinical parameters in PPS, lays the ground for treatment replication studies, with infrequent dosing intervals, as compared to many other chronic neuro-inflammatory conditions. If successful, there is an option for a relatively cheap, safe and effective treatment modality in this chronic condition which previously has lacked any options for immuno-modifying strategies.

## Authors’ contributions

HG conceived the study and contributed in the design, in patient evaluation, sample preparation, data acquisition, preparation and analysis. MK carried out the sample preparation and worked with the data acquisition, preparation and analysis. KB conceived the study and participated in the design, in patient evaluation, data acquisition, preparation and analysis. TO conceived the idea and helped with the data analysis. All authors contributed in the manuscript preparation, read and approved the final manuscript.

## References

[B1] Farbu E Gilhus NE Barnes MP Borg K de VM Driessen A EFNS guideline on diagnosis and management of post-polio syndrome. Report of an EFNS task force Eur J Neurol 2006 13 795 801 16879288 10.1111/j.1468-1331.2006.01385.x

[B2] Gonzalez H Olsson T Borg K Management of postpolio syndrome Lancet Neurol 2010 9 634 642 20494327 10.1016/S1474-4422(10)70095-8

[B3] Trojan DA Cashman NR Post-poliomyelitis syndrome Muscle Nerve 2005 31 6 19 15599928 10.1002/mus.20259

[B4] Grimby G Stalberg E Sandberg A Sunnerhagen KS An 8-year longitudinal study of muscle strength, muscle fiber size, and dynamic electromyogram in individuals with late polio Muscle Nerve 1998 21 1428 1437 9771666 10.1002/(sici)1097-4598(199811)21:11<1428::aid-mus10>3.0.co;2-x

[B5] McComas AJ Quartly C Griggs RC Early and late losses of motor units after poliomyelitis Brain 1997 120 Pt 8 1415 1421 9278631 10.1093/brain/120.8.1415

[B6] Bartholdi D Gonzalez H Borg K Melki J Absence of SMN gene deletion in post-polio syndrome Neuromuscul Disord 2000 10 99 10714583 10.1016/s0960-8966(99)00076-0

[B7] Cashman NR Trojan DA Correlation of electrophysiology with pathology, pathogenesis, and anticholinesterase therapy in post-polio syndrome Ann N Y Acad Sci 1995 753 138 150 7611623 10.1111/j.1749-6632.1995.tb27540.x

[B8] Dalakas MC Pro-inflammatory cytokines and motor neuron dysfunction: is there a connection in post-polio syndrome? J Neurol Sci 2002 205 5 8 12409176 10.1016/s0022-510x(02)00314-3

[B9] Rekand T Langeland N Aarli JA Vedeler CA Fcgamma receptor IIIA polymorphism as a risk factor for acute poliomyelitis J Infect Dis 2002 186 1840 1843 12447772 10.1086/345769

[B10] Sharief MK Hentges R Ciardi M Intrathecal immune response in patients with the post-polio syndrome N Engl J Med 1991 325 749 755 1651456 10.1056/NEJM199109123251101

[B11] Fordyce CB Gagne D Jalili F Alatab S Arnold DL Da CD Elevated serum inflammatory markers in post-poliomyelitis syndrome J Neurol Sci 2008 271 80 86 18474371 10.1016/j.jns.2008.03.015

[B12] Gonzalez H Khademi M Andersson M Wallstrom E Borg K Olsson T Prior poliomyelitis-evidence of cytokine production in the central nervous system J Neurol Sci 2002 205 9 13 12409177 10.1016/s0022-510x(02)00316-7

[B13] Gonzalez H Khademi M Andersson M Piehl F Wallstrom E Borg K Prior poliomyelitis-IVIg treatment reduces proinflammatory cytokine production J Neuroimmunol 2004 150 139 144 15081258 10.1016/j.jneuroim.2004.01.010

[B14] Farbu E Rekand T Vik-Mo E Lygren H Gilhus NE Aarli JA Post-polio syndrome patients treated with intravenous immunoglobulin: a double-blinded randomized controlled pilot study Eur J Neurol 2007 14 60 65 17222115 10.1111/j.1468-1331.2006.01552.x

[B15] Gonzalez H Ottervald J Nilsson KC Sjogren N Miliotis T Von BH Identification of novel candidate protein biomarkers for the post-polio syndrome - implications for diagnosis, neurodegeneration and neuroinflammation J Proteomics 2009 71 670 681 19100873 10.1016/j.jprot.2008.11.014

[B16] Kaponides G Gonzalez H Olsson T Borg K Effect of intravenous immunoglobulin in patients with post-polio syndrome – an uncontrolled pilot study J Rehabil Med 2006 38 138 140 16546773 10.1080/16501970500441625

[B17] Gonzalez H Sunnerhagen KS Sjoberg I Kaponides G Olsson T Borg K Intravenous immunoglobulin for post-polio syndrome: a randomised controlled trial Lancet Neurol 2006 5 493 500 16713921 10.1016/S1474-4422(06)70447-1

[B18] Werhagen L Borg K Pain in post polio syndrome - effects of intravenous immunglobulin treatment J Rehabil Med 2011 in press 10.2340/16501977-0884 22031351

[B19] March of Dimes March of Dimes International Conference on Post Polio Syndrome. Identifying Best Practices in Diagnosis and Care 2000 White Plains

[B20] McHorney CA Ware JE Raczek AE The MOS 36-Item Short-Form Health Survey (SF-36): II. Psychometric and clinical tests of validity in measuring physical and mental health constructs Med Care 1993 31 247 263 8450681 10.1097/00005650-199303000-00006

[B21] Ware JE Sherbourne CD The MOS 36-item short-form health survey (SF-36). I. Conceptual framework and item selection Med Care 1992 30 473 483 1593914

[B22] Bernklev T Jahnsen J Lygren I Henriksen M Vatn M Moum B Health-related quality of life in patients with inflammatory bowel disease measured with the short form-36: psychometric assessments and a comparison with general population norms Inflamm Bowel Dis 2005 11 909 918 16189421 10.1097/01.mib.0000179467.01748.99

[B23] Elliott TE Renier CM Palcher JA Chronic pain, depression, and quality of life: correlations and predictive value of the SF-36 Pain Med 2003 4 331 339 14750909 10.1111/j.1526-4637.2003.03040.x

[B24] Kosinski M Keller SD Ware JE Hatoum HT Kong SX The SF-36 Health Survey as a generic outcome measure in clinical trials of patients with osteoarthritis and rheumatoid arthritis: relative validity of scales in relation to clinical measures of arthritis severity Med Care 1999 37 MS23 MS39 10335741 10.1097/00005650-199905001-00003

[B25] Saba J Quinet RJ Davis WE Krousel-Wood M Chambers R Gomez N Inverse correlation of each functional status scale of the SF-36 with degree of disease activity in systemic lupus erythematosus (m-SLAM) Joint Bone Spine 2003 70 348 351 14563462 10.1016/s1297-319x(03)00065-4

[B26] Ware JE Kosinski M Gandek B Aaronson NK Apolone G Bech P The factor structure of the SF-36 Health Survey in 10 countries: results from the IQOLA Project. International quality of life assessment J Clin Epidemiol 1998 51 1159 1165 9817133 10.1016/s0895-4356(98)00107-3

[B27] Harada ND Chiu V Stewart AL Mobility-related function in older adults: assessment with a 6-minute walk test Arch Phys Med Rehabil 1999 80 837 841 10414771 10.1016/s0003-9993(99)90236-8

[B28] Steffen TM Hacker TA Mollinger L Age- and gender-related test performance in community-dwelling elderly people: six-minute walk test, berg balance scale, timed up & go test, and gait speeds Phys Ther 2002 82 128 137 11856064 10.1093/ptj/82.2.128

[B29] Price DD Bush FM Long S Harkins SW A comparison of pain measurement characteristics of mechanical visual analogue and simple numerical rating scales Pain 1994 56 217 226 8008411 10.1016/0304-3959(94)90097-3

[B30] Khademi M Kockum I Andersson ML Iacobaeus E Brundin L Sellebjerg F Cerebrospinal fluid CXCL13 in multiple sclerosis: a suggestive prognostic marker for the disease course Mult Scler 2011 17 335 343 21135023 10.1177/1352458510389102

[B31] Khademi M Illes Z Gielen AW Marta M Takazawa N Baecher-Allan C T Cell Ig- and mucin-domain-containing molecule-3 (TIM-3) and TIM-1 molecules are differentially expressed on human Th1 and Th2 cells and in cerebrospinal fluid-derived mononuclear cells in multiple sclerosis J Immunol 2004 172 7169 7176 15153541 10.4049/jimmunol.172.11.7169

[B32] Langrish CL Chen Y Blumenschein WM Mattson J Basham B Sedgwick JD IL-23 drives a pathogenic T cell population that induces autoimmune inflammation J Exp Med 2005 201 233 240 15657292 10.1084/jem.20041257 PMC2212798

